# Modification of everyday activities and its association with self-awareness in cognitively diverse older adults

**DOI:** 10.1371/journal.pone.0222769

**Published:** 2019-11-07

**Authors:** Danielle Shaked, Preeti Sunderaraman, Jennifer Piscitello, Sarah Cines, Christiane Hale, Davangere Devanand, Jason Karlawish, Stephanie Cosentino

**Affiliations:** 1 Cognitive Neuroscience Division of the Taub Institute for Research on Alzheimer’s Disease and the Aging Brain, Columbia University Medical Center, New York, NY, United States of America; 2 Department of Psychology, University of Maryland, Baltimore County, Baltimore, MD, United States of America; 3 Gertrude. H. Sergievsky Center, Columbia University Medical Center, New York, NY, United States of America; 4 Department of Neurology, Columbia University Medical Center, New York, NY, United States of America; 5 Division of Geriatric Psychiatry, Department of Psychiatry, Columbia University Medical Center and New York State Psychiatric Institute, New York, NY, United States of America; 6 Healthy Brain Research Center, University of Pennsylvania, Perelman School of Medicine, Philadelphia, PA, United States of America; Nathan S Kline Institute, UNITED STATES

## Abstract

Cognitive impairment (CI) in older adults is frequently accompanied by difficulty performing complex everyday activities (e.g., managing finances). However, it is unclear if and how older adults with CI modify their activities (i.e., Do individuals *continue*, *monitor*, *seek help with*, *change their approach to*, or *stop* different activities?). In the current study, we examined if older adults with CI are concerned about their ability to carry out complex activities, if and how they modify activities based on their concern, and the factors associated with activity modification. We hypothesized that older adults with CI will more frequently be concerned about, and modify, everyday activities than cognitively healthy (HE) older adults, and that higher awareness of memory loss in the CI group would relate to more frequent modification. The sample included 81 older adults (51 HEs; mean age 70.02 (7.34) and 30 CI; mean age 75.97 (8.12)). Compared to HEs, the CI group reported having more concern about, *F*(3,77) *= 5*.*50*, *p* = 0.02, and modifying a greater number of activities, *F*(3,77) *= 5*.*02*, *p* = 0.03. Medication management (30%) and completing taxes (33.3%) were among the most frequently modified activities for the CI and HE groups, respectively. In the CI group, higher memory awareness was associated with more concern (*r* = .53, *p* = .005) and activity modification (*r =* 0.55, *p =* .003). Findings provide novel information about how cognitively diverse older adults navigate complex activities in daily life. We propose a preliminary theoretical model by which self-awareness may influence navigation of everyday activities in the context of CI.

## Introduction

A diagnosis of dementia reflects the clinical determination that an individual’s cognitive deficits have significantly interfered with his or her ability to successfully carry out instrumental activities of daily living (IADLs) [1]. The nature and degree of functional changes in everyday activities has been documented extensively in individuals with Alzheimer’s disease (AD) and other dementias [2–6]. In contrast, the diagnosis of Mild Cognitive Impairment (MCI) requires the presence of both subjectively reported and objectively measured cognitive deficits which are not yet sufficient to significantly interfere with capacity for independence in everyday activities [7]. Nonetheless, there is evidence that subtle deficits in functional abilities such as shopping, driving, and financial management exist in the context of MCI [6, 8, 9], or even earlier than MCI [10, 11]. The time at which functional changes, and ultimately functional deficits, emerge in a given individual likely reflects a combination of factors including the degree and domain of cognitive impairment, the individual’s premorbid level of functioning, the presence of psychiatric or physical comorbidities, and the extent of support by other family members, among other things.

Despite the universal decline in everyday function that occurs as individuals move along the spectrum of cognitive aging from a state of health to conditions of MCI and dementia, and the negative outcomes associated with functional loss (i.e., motor vehicle accidents, financial mismanagement), little is known about how older adults including those with cognitive impairment manage functional difficulties. In the physical realm, modification of how tasks are performed (e.g., getting up from a chair) is associated with improved IADLs among frail older adults [12–15]. In the cognitive realm, older individuals are encouraged to use compensatory strategies for IADLs (e.g., pillboxes) to improve IADLs [16], and there is some evidence that individuals with MCI implement compensatory strategies (such as memory aids) more frequently than cognitively normal older adults [17]. However, whether older adults, both with and without cognitive impairment, modify everyday activities to address functional limitations more broadly remains to be elucidated, as does *how* they may modify activities. In particular, are they more likely to ask for help, change their approach, or stop the activity altogether? Determining the answers to these questions, as well as identifying the factors that influence how older adults navigate everyday life, may help mitigate the physical, psychosocial, financial, and societal costs of functional loss in MCI and dementia.

Intact metacognitive functioning, including awareness of one’s memory loss, is likely to be a particularly important determinant of whether individuals with cognitive impairment (CI) are concerned about and/or modify their approach to everyday activities in the face of cognitive and functional changes. It is well known that a large proportion of individuals with early AD [18–21] and even MCI [22, 23] have reduced awareness of their cognitive symptoms. Although awareness decreases on average over the entire disease course, research suggests that there is no substantial difference in the degree of awareness across MCI and mild AD [23–25]. In fact, in both groups, there is considerable heterogeneity in awareness [23, 26, 27].

Previous work from our lab showed that among individuals with AD, those with reduced memory awareness were less likely to appreciate the need for medication management strategies than those who are aware of their memory changes [28]. Failure to modify one’s approach to medication management and other cognitively complex activities could have negative consequences. Indeed, there is evidence that individuals who are unaware of their memory loss are more likely to engage in dangerous behaviors such as leaving the stove on, driving, and poor medical adherence [29, 30].

**Fig 1:**
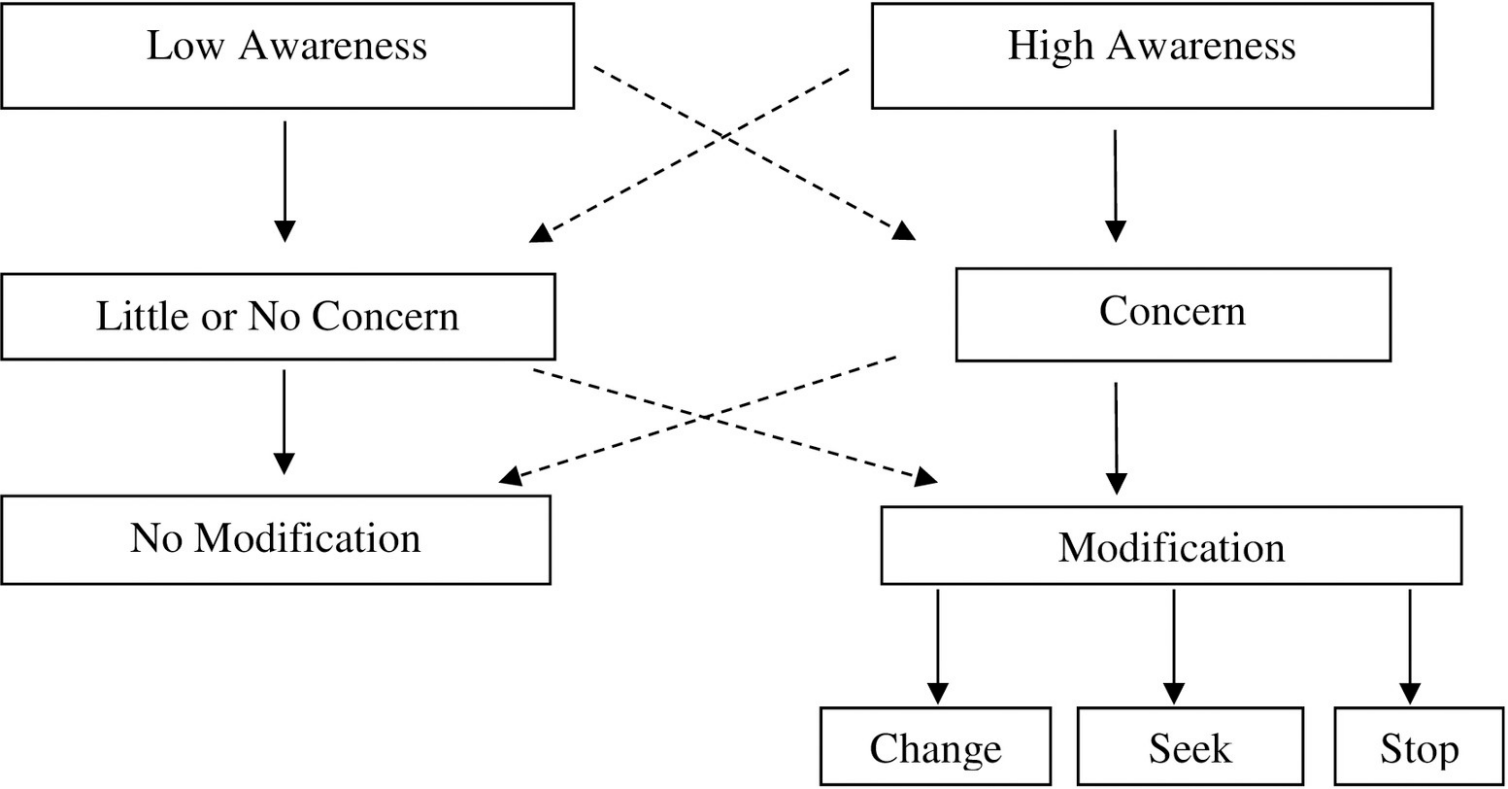
Model of awareness and behavior modification.

Fig 1 depicts a potential model by which memory awareness and behavioral modification may interface. In the primary paths of the model marked by the solid arrows, lower levels of awareness are hypothesized to result in a relative lack of concern regarding the ability to carry out everyday activities, and consequently low levels of behavior modification. On the other hand, individuals with high levels of awareness, are hypothesized to have greater levels of concern about their ability to navigate complex activities, and therefore be more likely to engage in modification, such as changing the way an activity is done, seeking help to accomplish that activity, or discontinuing the activity altogether. While the current study does not aim to explore all aspects of this model, as a first step, it seeks to investigate the association between awareness, concern, and modification.

To our knowledge, there are no existing studies examining the decisions that cognitively impaired older adults make about how to carry out everyday activities, or the extent to which reduced memory awareness influences those decisions. The aim of the current study was therefore to survey decisions about a range of everyday activities among individuals across the spectrum of cognitive aging, including those with MCI and dementia as well as cognitively healthy older adults. We hypothesized that among individuals with cognitive impairment (MCI and AD), those with greater memory awareness would more frequently modify their approach to cognitively demanding everyday activities.

## Materials and methods

### Participants

Eighty-one older adults with a range of cognitive functioning, including 51 cognitively healthy older adults, 24 individuals diagnosed with MCI, and 6 individuals diagnosed with dementia (Probable or Possible Alzheimer’s disease [AD]), were enrolled from ongoing studies of cognitive aging. Individuals were recruited into the current study as healthy controls if they performed above standard cutoffs on a cognitive screener, including the Dementia Rating Scale [31] or Mini-Mental State Examination (MMSE; [32]), and were free of neurological or significant psychiatric disease. Individuals were considered to be cognitively impaired if they met criteria for MCI or AD (probable or possible). Diagnoses of AD were made according to the Neurologic Disorders and Stroke-Alzheimer’s Disease and Related Disorders Association (NINDS-ADRDA) criteria. Only patients with mild dementia, defined as a score of 19 or greater on the Mini-Mental State Examination (MMSE) [33] and Clinical Dementia Rating (CDR) Scale = 1 [34] were recruited for this study. Diagnoses of MCI were based on the report of cognitive decline by the patient or an informant, in conjunction with objective impairment on standard neuropsychological testing, and lack of significant functional impairment (CDR = 0.5) [7]. All participants provided written consent and the procedures described above were approved by Institutional Review Board of Columbia University Medical Center and University of Pennsylvania. Capacity to consent was determined at the time of informed consent. As individuals with only mild degrees of cognitive impairment were included in this study, the vast majority were judged to have capacity to consent. If individuals were judged not to have the capacity to consent, knowledgeable informants provided informed consent along with the participants assent. Details regarding the individual cognitive tests used for diagnosis in each referring study, and agreement on case classification have been published previously [35–38]. Individuals with ongoing psychiatric conditions or a history of head injury, stroke, and other neurologic illnesses that may affect cognition or the presentation of MCI or dementia were excluded. To increase our statistical power, we combined individuals with MCI and AD into a single cognitively impaired group.

### Measures

#### Clinical Ratings of Awareness (CRA)

Examiners conducted brief interviews with participants with MCI and dementia to determine their level of awareness regarding memory deficits. Examiners assigned scores ranging from 1 to 4 using a modified version of the Anosognosia Rating Scale [21]. Participants were rated as follows: 4 = Full Awareness (endorsement of memory loss along with the recognition that the loss is consequential and/or abnormal); 3 = Moderate Awareness (endorsement of memory loss; however, loss is discussed in the context of “normal” age related changes); 2 = Shallow Awareness (inconsistent or transient recognition of memory loss); 1 = No Awareness (matter-of-fact denial of memory impairment). This instrument has been shown to correlate with patient report of memory functioning in a closed-ended format, as well as with objective scores of memory monitoring, and to have high inter-rater reliability [28, 39].

#### Activity modification

Participants were queried with regard to 12 everyday activities. The specific instructions were: “*Below is a series of questions about different activities in your life*, *like taking medications and paying bills*. *We are interested in knowing if you have changed your approach to any of these activities based on concerns about changes in your memory or other thinking abilities*.*”* Three of the activities related to financial matters (i.e., balancing a checkbook, doing taxes, paying bills), three related to household management (i.e., cooking, grocery shopping, caring for grandchildren), three related to commuting (i.e., traveling to a new destination, driving, utilizing public transportation), two related to personal care (i.e., medication management, scheduling appointments), and one related to employment (i.e., working). Response choices are outlined in Table 1. Individuals were instructed to select a single response per item. Although an informant version of this scale is in development, the current study sought only to examine the participant’s perceptions and decisions.

**Table 1:** Table detailing the response choices and the derived primary outcome scores from the Activity Modification scale.

Response Choices		Modification Score	Concern Score	Concern without modification
1	I have or had concerns so I changed the way I do this activity	1	1	0
2	I have or had concerns, so someone helps me with this activity			
3	I have or had concerns so I stopped doing the activity completely			
4	I have or had concerns, but I have not changed the way I do this activity	0		1
5	I have no concerns about my ability to do the task independently		0	0
6	I never really did this activity			

Scoring: Item scores (0 or 1) were summed across all 12 items to create three separate primary outcome scores ranging from 0–12, as outlined in Table 1 and described here: (1) *Modification*: Total number of activities an individual endorsed modifying, either by changing one’s approach to an activity, getting help, or discontinuing the activity (Table 1; responses 1–3), (2) *Concern*: Total number of activities about which an individual endorsed concern, irrespective of modifying or not modifying the activity (Table 1; responses 1–4), and (3) *Concern without Modification*: Total number of activities about which an individual endorsed concern without modifying the behavior (Table 1; response 4). This item is examined as a primary outcome because it is the only item that reflects a discordance between concern and modification.

To account for activities that an individual may have never performed (Table 1; response 6), an overall score for *Never Did Activity* was obtained across the 12 items (0–12). Each of the three Primary Outcome scores were then calculated as a percentage of the total number of items that the participant had engaged in (12 –*Never Did Activity* score). For example, if an individual endorsed modifying 1 out of 12 activities, but endorsed never performing 8 activities, then the percent modification score was 1/(12–8) = 0.25. In other words, out of the four activities that this individual has performed, only one was modified (25%). The entire range of points for percent modification was thus 0–100% across the 12-item scale, with higher scores indicating more modification. For statistical analyses, these three % Primary Outcome scores–% Modification, % Concern and % Concern without Modification–were used.

In addition to calculating the % Primary Outcome scores, we also examined the relative frequency with which participants employed *specific types of modification*. For example, if an individual endorsed modifying six activities (three that were changed and three that were stopped), the *% change* score and *% stop* scores would each be 3/6, or 50%. For statistical analysis, % Modification Type scores–% seek help, % change, and % stop–were used.

#### Cognitive Assessment

The MMSE and a list-learning test (either the Selective Reminding Test or the Philadelphia Verbal Learning Test) was administered as part of the screening process by a few of the referring studies. We obtained this data wherever possible, and used the total MMSE score and a memory retention score ((Delayed Recall / Immediate Recall)*100) for our analysis.

### Procedures

Individuals underwent diagnostic testing through multiple ongoing studies of cognitive aging prior to participating in this study. For the current study, participants completed the MMSE, and those who carried diagnoses of MCI or dementia also received clinical ratings of awareness. Clinical ratings of awareness were not obtained on healthy elders as these ratings were designed to capture awareness of clinically diagnosed memory loss. All participants then completed the Activity Modification scale. All participants provided written consent and the procedures described above were approved by the Institutional Review Board of Columbia University Medical Center and University of Pennsylvania.

### Data analysis

Chi square analyses and independent samples t-tests were used to examine differences in demographic variables and MMSE across the healthy elder (HE) and CI groups. Given the demographic differences in age and gender across the two groups, ANCOVAs adjusting for these two variables were conducted to examine differences in Primary Outcome scores (*Modification*, *Concern*, and *Concern without Modification*) across groups (see S1 File for analysis using age-, gender- and education-matched groups). To determine the extent to which results were influenced by responses from individuals who engaged in relatively few (<2/3) of the listed activities, ANCOVAs were rerun after excluding such participants (see S1 File). To determine whether the HE and CI groups employed different types of activity modification, a mixed model ANOVA was applied with group as the between-factor variable and *% Modification Type* (help, change, stop) as the within subject factors. Data were log transformed to reduce skewness for this analysis. Finally, Spearman correlations were used to examine the association between the *% Modification Type* score and participant characteristics. We also ran correlations between % Modification Type and % memory retention.

## Results

### Descriptive information

Descriptive information regarding demographic characteristics and performance on each of the measures is outlined in Table 2 as a function of diagnostic group. Within the CI group, there were no differences in age *t*(28) *=* 0.88, *p =* 0.38, education *t*(28) *=* 0.77, *p =* 0.45, MMSE *t*(28) *=* 0.77, *p =* 0.45, or memory awareness *t*(28) *=* -0.72, *p =* 0.48 between participants with MCI and dementia. It is possible that the lack of demographic differences seen across groups is due to sample size, and that with more statistical power, between group variability would have been observed. As expected, the CI group achieved lower scores on the MMSE (range: 10–30) than the HE group (range: 26–30), *t*(43) *=* 3.96, *p<0*.*001*. Differences were also found for age, *t*(79) = -3.38, *p =* 0.001, and gender, *χ*^*2*^ (1, *N* = 81) = 5.87, *p* = 0.02, but not education *t*(78) *=* 0.96, *p =* 0.34 or ethnicity, *χ*^*2*^ (1, *N* = 81) = 0.05, *p* = 1.00. To consider demographic differences, subsequent analyses comparing the HE and CI groups on the primary outcomes of interest were adjusted for age and gender. Mean scores for each of the Primary Outcomes (*Modification*, *Concern*, and *Concern without Modification*) are presented by group in Table 3, along with mean scores for *Not Concerned* and *Never Did Activity*.

**Table 2:** Descriptive information about demographics for all groups.

	HE (N = 51)	MCI (N = 24)	Dementia (N = 6)	CI (N = 30)
Age, mean (SD)	70.02 (7.34)	76.63 (7.52)	73.33 (10.58)	75.97 (8.12)
Sex, n (%)				
Female	36 (70.6)	9 (37.5)	4 (66.7)	13 (43.30)
Race, n (%)				
Caucasian	38 (74.5)	18 (75)	5 (83.3)	23 (76.7)
African American	13 (25.5)	4 (16.7)	1 (16.7)	5 (16.7)
Asian	0 (0)	1 (4.2)	0 (0)	1 (3.3)
Native Indian	0 (0)	1 (4.2)	0 (0)	1 (3.3)
Education, mean (SD)	16.28 (2.21)	15.92 (3.61)	14.67 (3.26)	15.67 (3.53)
MMSE, mean (SD)	29.33 (1.39)^a^	25.04 (4.71)	23.50 (2.51)	24.73 (4.37)
% Retention Score	NA	39.42 (27.25)	25 (35.36)	38.04 (27.38)^b^
Awareness, mean (SD)	NA	2.71 (0.85)	3.00 (.89)	2.78 (0.85)
Awareness, n %				
Full awareness (4)	NA	3 (12.5)	2 (33.3)	5 (16.7)
Moderate awareness (3)	NA	11 (45.8)	2 (33.3)	12 (43.3)
Shallow Awareness (2)	NA	5 (20.8)	2 (33.3)	7 (23.3)

Note: HE = Healthy Elder; MCI = Mild Cognitive Impairment; Dementia = Probable and Possible AD; CI = Cognitively Impaired; MMSE = Mini-Mental State Examination; NA = Not Applicable.

^a^n (for HE) = 15 and n (for CI) = 45; Significant difference across HE versus CI group.

^b^n (for CI) = 21.

**Table 3:** Mean scores on the Activity Modification scale.

	Healthy Elders	Cognitively Impaired
Never Did Activity	1.39 (1.04)	2.5 (2.10)
Not Concerned	9.31 (2.06)	6.93 (3.61)
Primary Outcome Scores		
Modification	1.04 (1.60)	2.1 (2.56)
Concern	1.29 (1.89)	2.57 (3.02)
Concern without modification	0.25 (0.66)	0.47 (1.33)

Note. Range for each score is 0–12.

### Primary outcomes

After adjusting for age and gender, compared to the HE group, the CI group had higher % *Modification*, *F*(3, 77) *=* 5.02, *p* = 0.03, partial *η^2^* = .01 and higher *% Concern* than the HE group, *F*(3, 77) *=* 5.50, *p* = 0.02, partial *η^2^* = .07. There was no difference between the two groups for *% Concern without Modification*, *F*(3, 77) = .97, *p* = 0.33, partial *η^2^* = .01.

With respect to the *type of modification* between the two groups (Table 4), results from the mixed model ANOVA revealed no main effects of *% Modification Type*, *F*(2,78) = .88, *p* = .42, partial *η^2^* = .02 or group, *F*(1, 39) = .59, *p* = .45, partial *η^2^* = .01 and no significant *% Modification Type* x group interaction, *F*(2,78) = .19, *p* = .82, partial *η^2^* = .01. Results were identical when analysis for the Primary Outcomes and *type of modification* was rerun when the HE and CI groups were demographically matched (see S1 File).

**Table 4:** Modification type (%) by group.

	Healthy Elders	CI [± 95%]	Cognitively Impaired	CI [± 95%]
Help	43.5	26.4–60.6	48.5	28.6–68.3
Stop	19.6	6.0–33.2	14.7	2.2–27.3
Change	36.9	21.0–52.8	36.8	16.8–56.8

With regard to the specific activities most frequently modified, in the CI group, 30% (*n* = 9) modified their approach to managing medications, and 26.7% (*n = 8*) modified completing taxes, driving, and traveling to new destinations. Among the HE group, 33.3% (*n* = 17) modified their approach to completing taxes, and 11.8% (*n* = 6) modified travelling to new destinations.

### Correlates of activity modification outcomes

Among the entire sample, all primary outcomes, ***%*** *Modification* (*r* = -.32, *p* = .03), % *Concern without modification* (*r* = -.50, *p* < .001), and % *Concern* (*r* = -.40, *p* = .006) were inversely associated with global cognition. Education was inversely associated with *Concern without modification* (*r* = -.25, *p* = .026). No other associations were significant, including those for age and gender. As hypothesized, among the CI group, awareness was positively correlated with *Modification* (*r* = .55, *p* = .003) and *Concern* (*r* = .53, *p* = .005), such that those with higher memory awareness modified more activities and expressed more concern. Memory was not associated with any of the primary outcomes; ***%*** *Modification* (*r* = .26, *p* = .25), % *Concern without modification* (*r* = -.38, *p* = .08), and % *Concern* (*r* = .16, *p* = .48)

## Discussion

Successful navigation of everyday life requires an individual to execute a series of cognitively demanding activities. Age-related cognitive changes, particularly those associated with MCI or dementia, require individuals to evaluate their ability to carry out such activities, and possibly modify their approach to engaging in these activities. The current study examined the extent to which cognitively diverse older adults, including a mixed group diagnosed with MCI or dementia, modified their approach to a range of everyday activities, and the extent to which memory awareness was associated with activity modification.

### Summary of results

The CI group expressed concern about more activities than the healthy older adults, and correspondingly, modified a greater number of everyday activities. There was no significant difference in the type of modification used in either group (e.g., seek help versus stop an activity). Qualitative examination of the specific activities that were modified most frequently in each group, however, revealed some differences. For example, both driving and medication management were among the most frequently modified activities in the cognitively impaired group, whereas completing taxes was most frequently modified among the healthy group. Finally, with regard to the demographic correlates of activity modification, those with lower education reported more frequent concern about their ability to perform the activity without modifying their behavior.

With regard to the nature of modifications (seek help with *versus* change *versus* stop), individuals endorsed seeking help more frequently than the other two options. A few previous studies have noted that individuals with MCI and dementia tend to use various external and internal coping strategies such as use of memory aids, frequent repetition, engaging in deliberate, systematic thinking, and slowing down their pace when engaging in everyday activities [17, 40, 41]. It has been speculated that the nature of modification may follow a hierarchical pattern, as one study implied that individuals may at first attempt to change their behaviors by trying out various strategies, before requesting help from others or stopping the behavior altogether, depending on the nature of the task [40]. This possibility needs to be explored by future longitudinal studies and more data are needed to test the conceptual framework of the model outlined above. Perhaps another possibility to be tested is whether the nature and the timing of the modification is related to the amount of social support and resources that are available to the individuals in their environment.

Qualitative analyses revealed that the specific activities modified most frequently were different across groups, potentially reflecting progressive decline in activities that may become more challenging to perform as pathology manifests in older adults. In our study, we found that the top activity modified by the CI group pertained to medication management whereas for the HE group it was completing taxes. While both groups endorsed modifying their approach to similar activities such as traveling to new destinations and driving, the pattern differed across the groups. For example, whereas 33.3% (n = 17) HE’s modified the way they did their taxes, only 26.7% (n = 9) of CI endorsed this item. Thus, it seems that the CI group modification of activities may be a manifestation of pathology-related changes in behaviors. Dissociations in the types of activities about which individuals are concerned and modify could ultimately have relevance for identifying typical versus pathological aging. Although intriguing, the changes in pattern and range of activities should be explored more exhaustively across larger and heterogeneous samples of older adults.

### Awareness and activity modification

It has long been known that a proportion of individuals diagnosed with MCI or dementia are unaware of their symptoms [39, 42, 43], yet only a handful of studies have investigated the manner in which unawareness relates to everyday decisions. For example, previous work has shown that reduced awareness is associated with resistance to discontinuing driving [44], and decreased appreciation for the need to implement medication management strategies [45]. The current study extends these findings to demonstrate that lower memory awareness relates to reduced concern about, and modification of, everyday activities. As shown in Fig 1 and as our results suggest, lower levels of awareness may result in a lack of concern about one’s ability to navigate everyday activities, which in turn makes individuals less likely to modify those activities. Concurrently, those with high levels of awareness are more likely to make behavioral modifications, as they are more concerned about their ability to appropriately carryout activities.

Certainly, the relationship between each level of the model (Fig 1; i.e., awareness, concern, and modification) may not be straightforward, as indicated by the dashed pathways in the figure. It is possible that in the context of low awareness, individuals may still feel concerned about their ability to carry out complex activities, based on premorbid personality characteristics, feedback from others, or an implicit level of awareness regarding one’s own difficulties [46]. Conversely, those with high levels of awareness may be unconcerned about their ability to carry out activities in the event that awareness of cognitive deficit is accompanied by indifference (i.e., anosodiaphoria).

Finally, it is possible that in the two lower levels of the model (Fig 1), concern and modification are not linked in a uniform way. That is, a lack of concern need not preclude behavioral modification (i.e., modifying behavior for another reason), and the presence of concern need not necessitate behavioral modification. Indeed, chi-square analysis (results not significant) revealed that individuals in the CI group (23.3%; n = 7) as well as the HE group (15.7%; n = 8) reported having concerns about certain activities without modifying their behavior. As there was no difference across groups in the degree of concern without modification, it may not be an abnormal pattern of behavior. It is possible that these individuals accurately sense that their difficulty is not great enough to require modifying their behavior. The finding that fewer years of education was associated with more instances of concern without modification could also indicate that the decision to modify behavior may reflect health literacy, or the availability of resources to engage in behavioral modification, such as buying a memory aid [47–49].

It is also possible that there are heterogeneous reasons for endorsing concern without modification, and that in the context of MCI and AD, individuals fail to make behavior changes despite accurately perceiving that a change is warranted. Indeed, ‘dissociation between knowing and doing’ [50] has been well documented in patients with brain injury, and highlights the distinction between knowing what to do (hypothetically) and the ability to act on it (actual behavior based on the knowledge). This dissociation maps onto the two primary components of metacognition, monitoring and control [39, 51]. Whereas monitoring comprises the ability to evaluate one’s own cognition and behavior, control relates to the actions that are taken based on the information that is being monitored. Clear dissociations between these two constructs have been observed not only in patients with brain injury, but also in those with schizophrenia [52, 53]. In the case of MCI and dementia, compromised ability to implement behavior modification might reflect a dysfunctional control system in the presence of intact monitoring, potentially due to memory deficits (i.e., “forgetting” to modify the behavior), executive function deficits (e.g., mental inflexibility), affective changes (e.g., apathy) or some other factor. In individuals with cognitive impairment, functional deficits may be most easily mitigated in the context of intact monitoring, particularly when control processes are also intact.

The current paper was not designed to fully test the outlined model (Fig 1), or to explicate each of the various pathways, and future work is needed in this regard. However, this model offers a preliminary conceptual framework within which to consider the aims and results of the current study, and to guide future studies. Perhaps most importantly, this model illuminates potential pathways between metacognition and everyday behavior in the context of cognitive aging, facilitating consideration of traditionally “cognitive” constructs such as awareness in the context of everyday, “real word” actions and decisions.

Findings from the current study emphasize the clinical and practical relevance of disordered memory awareness or self-awareness, often referred to as anosognosia. In the context of reduced awareness, treatment professionals and family members should play an active part in everyday decision making, and evaluate the need for checking and/or supervising patient activities. Based on the specific context, behavioral management strategies and environmental adaptations geared towards monitoring financial resources, recruiting a reliable money manager, nurse and/or a home health aide, arranging for transportation, and/or referral to social services can be discussed. In addition, it may be valuable to provide educational and support resources to patients and thereby increase the likelihood of successfully engaging in modified behaviors. In the current study, preparing taxes, traveling to new destinations, driving, and managing medication were the activities most frequently modified by the CI group, suggesting that caregivers and participants alike may benefit from consideration of function in each of these areas, and discussing strategies to handle such changes. The fact that greater awareness is linked to greater activity modification is encouraging, however. It indicates that a person who is aware of their memory loss is likely to adjust their behavior in the context of cognitive changes (e.g., hiring a financial consultant, discontinuing driving). From a therapeutic perspective, this finding implies that by training individuals to become more aware of deficits, they may be able to automatically engage in various forms of activity modifications [54–56].

Of note, the Activity Modification scale used in the current study shares features with functional scales such as the *Lawton-Brody* Instrumental Activities of Daily Living *Scale* (IADL; [57]); however, the purpose of these scales differs in important ways. The Lawton-Brody IADL scale and other functional measurements are broadly designed to capture an individual’s functional level for each activity (i.e., unable to do the activity, able to do the activity with help, and does the activity independently). In contrast, the purpose of the current work was to assess decisions about modifying everyday activities in the context of functional or anticipated functional impairment. At any given level of functioning, self-awareness is likely to play a critical role in the decisions individuals make about everyday activities. Thus, while two individuals might have similar functional limitations in driving, for example, an individual who is aware of this limitation is more likely to modify their approach to the activity, and presumably reduce the likelihood of negative consequences. While data from the current study support this hypothesis, future work is needed to address the extent to which decisions about behavior modification are linked with negative functional outcomes.

### Limitations

There are several limitations to our study. Given the relatively small sample size, replication of these findings in a larger sample is warranted. Characterization of worried or concerned status among the HE group would have increased available data for the current analyses; future work might seek to quantify a base rate of concern among HEs as well as CI groups, and then examine the extent to which both groups may differentially engage in activity modification. Furthermore, future analyses should examine differences in activity modification across MCI and dementia groups, as these groups are likely to have both qualitative and quantitative differences related to activity modification. Finally, it is important to acknowledge that this is an exploratory study that did not seek to extensively characterize the psychometric properties of this measure. The task is meant to provide a descriptive assessment of activity modification in our sample, but is not fully validated and ready for use in a clinical setting. Ongoing work is expanding the repertoire of response choices available and number of activities assessed. For example, individuals may modify their behavior before it reaches a critical threshold for concern and, therefore, one may consider adding the category of modification without concern.

Once the task has been fully developed, work is needed to characterize the reliability and validity of this measure. It is worth emphasizing that within a given diagnostic stage (i.e., MCI or mild AD) we would not necessarily expect convergent validity between activity modification and cognitive or functional measures. Indeed, in this paper we did not find an association between memory performance and activity modification among the CI group although future studies should specifically investigate aspects of cognition that may relate more directly to activity modification (e.g., planning, and decision-making). Moreover, at any given level of functional ability, different individuals will *vary* in whether they modify their approach to an activity. We therefore would *not* expect a strong association between function and activity modification at a given cognitive level (e.g., MCI, mild dementia). For example, an individual might endorse difficulty managing medications on a functional scale, and at the same time endorse modifying their approach to managing medications by using a pillbox. While it is possible that this modification improves function, this may not necessarily be reflected on a functional scale wherein the person might still endorse having difficulty managing medications on a functional inventory. Inclusion of informant ratings will be critical for establishing these associations.

## Supporting information

S1 File (DOCX)

S2 File (SAV)
