# Supplementary material for: Modification of everyday activities and its association with self-awareness in cognitively diverse older adults
Source: PLoS One. 2019 Nov 7;14(11):e0222769. doi: 10.1371/journal.pone.0222769 (PMC6837494; doi:10.1371/journal.pone.0222769)
Supplement: S1 File — (DOCX) [file pone.0222769.s001.docx]

**Supplementary Data**

**1.** To determine the extent to which results were influenced by responses from individuals who engaged in relatively few (<2/3) of the listed activities, ANCOVAs were rerun after excluding such participants First, we examined the frequency of this response in each participant (see Supplementary Table 1). Analyses revealed that 86% of participants did at least 9 of the 12 activities included in the scale, while in contrast, 14% of participants endorsed never doing 4 or more activities. The activity that was most frequently endorsed as “Never Did” was Taking Care of Grandchildren (n=42).

| **Supplementary Table 1: Frequency of Endorsing “*Never Did the Activity*”** | | |
| --- | --- | --- |
| Total Number of “*Never Did the Activity*” Responses | N  (Total = 81) | Cumulative Percent |
| 0 | 13 | 16 |
| 1 | 29 | 52 |
| 2 | 23 | 80 |
| 3 | 5 | 86 |
| 4 | 5 | 93 |
| 5 | 2 | 95 |
| 6 | 3 | 98 |
| 8 | 1 | 100 |

In order to consider the effects of including individuals who endorsed never doing at least 4 activities (a third of the scale), we ran an ANCOVA including only individuals who completed at least 8 activities. Results were unchanged from the original analysis, although significance levels were somewhat attenuated which can be partly attributed to the reduced sample size. Specifically, the results indicated that the CI group had higher *% Concern* scores than the HE group, *F*(3, 66)*=4.26*, *p*=0.04, and the results were trending for higher % *Modification* scores, *F*(3, 66)*=3.49*, *p*=0.06. There was no difference between the two groups for *% Concern without Modification*, *F*(3, 66)=1.42, *p*=0.24.

**2.** We matched the HE and CI groups by age, gender and education and reran the analysis. The pattern of results is identical to the original findings. Specifically, for the primary outcomes, the CI group had higher % *Modification* scores, *t*(58)*=-2.80*, *p*=0.007, Cohen’s d = -0.77 and higher *% Concern* scores than the HE group, *t*(58)*=-3.00*, *p*=0.004, Cohen’s d = -0.74. There was no difference between the two groups for *% Concern without Modification*, *t*(58)*=-1.26*, *p*=0.21, Cohen’s d = -0.33. With respect to the *type of modification* between the two groups, results from the mixed model ANOVA revealed no main effects of *% Modification Type*, *F*(2,60) =2.83, *p*=0.07, partial η² = .09 or group, *F*(1, 30)=2.62, *p*=0.12, partial η² = .08 and no significant *%* *Modification Type* x group interaction, *F*(2,60) =0.21, *p*=0.81, partial η² = .01.
